# A ChIP on the shoulder? Chromatin immunoprecipitation and validation strategies for ChIP antibodies

**DOI:** 10.12688/f1000research.6719.1

**Published:** 2015-07-13

**Authors:** Fiona C. Wardle, Haihan Tan

**Affiliations:** 1 Randall Division of Cell and Molecular Biophysics, New Hunt's House, King's College London, Guy's Campus, London, SE1 1UL, UK

**Keywords:** Chromatin immunoprecipitation, antibody, validation, transcription factor, modified histone

## Abstract

Chromatin immunoprecipitation (ChIP) is a technique widely used in the study of epigenetics and transcriptional regulation of gene expression. However, its antibody-centric nature exposes it to similar challenges faced by other antibody-based procedures, of which the most prominent are issues of specificity and affinity in antigen recognition. As with other techniques that make use of antibodies, recent studies have shown the need for validation of ChIP antibodies in order to be sure they recognize the advertised protein or epitope. We summarize here the issues surrounding ChIP antibody usage, and highlight the toolkit of validation methods that can be employed by investigators looking to appraise these reagents.

## Introduction

Chromatin immunoprecipitation (ChIP) is a technique that has revolutionized our ability to identify regulatory sequences and epigenetic marks in the genome, and in doing so decipher networks of gene expression regulation that drive cell identity during development, disease, regeneration and evolution. It is used to determine if a protein of interest binds to, or is localized at, a specific DNA sequence. For example, it can be used to show where transcription factors and modified histones bind to a particular region of DNA in particular cells, allowing the identification of functional genomic sequences (e.g. 1).

Originally developed in bacterial cells ^2^ but soon applied to other cell and tissue types, particularly *Drosophila* cells and embryos (e.g. 3– 6), ChIP methods initially identified DNA bound by a protein of interest via a candidate approach for genomic regions suspected to interact with the protein, or by cloning and sequencing of immunoprecipitated DNA ^3– 6^. Over more than a decade, these methods allowed many regulatory regions to be identified, but it has been with the invention of genome-scale methods, such as microarrays (ChIP-chip) and more recently next generation sequencing (ChIP-seq; ^7– 13^), that the use of this technique has greatly expanded and allowed whole genome regulatory landscapes to be uncovered.

ChIP is now routinely used to map the genomic distribution of transcription factors, chromatin remodelling factors, and histone modifications in numerous cell types and organisms, and it is a technique central to the efforts of large-scale genomics consortia to map the regulatory genome. Indeed, the ENCODE and modENCODE communities used ChIP-seq in more than 100 cell types in mouse, human, *Drosophila* and *C. elegans* to map binding of over 140 DNA-interacting factors ^1, 14– 17^.

However, this technique depends on the use of antibodies to recognize the target protein of interest, and as with all techniques that rely on antibodies, issues of specificity and affinity arise ^18^. There is a growing realization among the scientific community that not all antibodies work as advertised, with problems of antibody specificity and variability causing projects to stall and published results to be irreproducible (discussed in 18, 19). Due to these problems there have been recent calls to standardize antibody manufacture, validation and reporting in publications (e.g. 19– 23). In this review, we will highlight some of the concerns and challenges that arise in selecting and validating antibodies for ChIP; we also discuss the need for validation standards, and highlight the validation guidelines used by ENCODE, modENCODE and other animal genome annotation consortia as a minimum standard for ChIP assays ^24^.

## ChIP method overview

ChIP usually involves lightly fixing cells of interest, usually with formaldehyde, to cross-link proteins and DNA. An alternative is native ChIP where a cross-linking reagent is not used. Chromatin is isolated from these cells and fragmented into pieces, usually in the range of 200–500 base pairs. This fragmentation may be enzymatic, e.g. with micrococcal nuclease, or mechanical, e.g. by sonication. An antibody recognizing the protein of interest, coupled to beads or other solid support, is then used to purify the protein, with its attached DNA, away from the rest of the sample. The cross-links, if present, are reversed and proteins in the sample are then degraded, leaving purified DNA that was associated with the protein of interest. Typically nowadays, this DNA is analysed either by high throughput sequencing to identify the genomic regions associated with the protein of interest, or by PCR with specific primers if binding sites are already known.

This procedure is a fairly involved process and the outcome is critically dependent on the quality of the antibody used – both its specificity and affinity for the protein of interest. Therefore like other procedures involving antibodies, it is crucial to validate antibodies used in ChIP to be confident of the results obtained. Many of the challenges faced in validating antibodies for other procedures are similar for ChIP, although there are issues unique to this protocol ( Box 1).

**Box 1. Potential concerns in antibody selection for ChIP assays**

- Commercially available ChIP-grade antibodies may not be validated between lots. They may also non-specifically cross-react with similar proteins, or not be validated in the organism or cell type of interest.

- Validation of non-ChIP-grade antibodies may be challenging, costly, and time-consuming.

- The choice of monoclonal antibodies recognizing only one epitope, or of polyclonal antibodies raised only to a part of the target protein, may reduce pull down of the target protein.

- The choice of polyclonal antibodies results in production of limited quantities of serum/antibody.

- Tagging of target proteins for tag-based pull downs may interfere with endogenous protein function.

- The straight overexpression of tagged proteins may result in spurious DNA binding.

- Antibodies validated as specific for the target protein may not bind to the target with high affinity in ChIP.

## Selecting an antibody

The choice of antibody for ChIP will depend on the target protein of interest and the antibodies that are already available. It may be that the protein of interest is well-studied, and that well-characterized antibodies are commercially available, which have been used and previously validated in ChIP in the cells of interest. In this case, little or no additional validation may be required. Indeed with the surge in the use of ChIP methodologies, many companies now sell ChIP-validated antibodies. This situation is most common for antibodies against histone modifications and dozens of companies offer such ChIP-validated antibodies (e.g. 25).

However, even with these ‘validated’ antibodies, it is critical to confirm what validation assays have been performed, and whether cross-reactivity has been reported. As an example, Egelhofer and colleagues tested over 200 commercially available antibodies raised to different histone modifications and found that more than 25% were not strictly specific to the modification advertised ^26^. This may in part be due to different lots of the same antibody differing considerably in their specificity ^26^, so it is crucial to know which lot(s) any validations have been performed on, and whether the current lot in hand has been tested.

Unfortunately, not all companies repeat validation tests on new lots ^27^, and so the user may need to validate the antibody themselves (more of which below). This issue also highlights the need for authors to include the lot number, company, and catalog number in materials and methods sections, when publishing papers in which antibodies are used. In addition, if the antibody has not been validated in the cells of interest, it should be validated in those specific cells before experimental use, since it may not behave in the same way as in tested cells ^24^.

It may, on the other hand, be the case that the ChIP will be against a protein for which no validated antibody has been produced. This is especially pertinent in the case of non-mammalian tissues, such as zebrafish or *Xenopus*, or if investigating a protein that has not received much attention in the past. In these situations, commercially available antibodies raised against homologous proteins from different species, or custom-manufactured antibodies, will have to be tested. Unfortunately, there are no hard and fast rules defining an archetypal antibody best for ChIP; however, we set out below some of the advantages and disadvantages of different types of antibodies.

## Monoclonal vs polyclonal

ChIP can be performed using either monoclonal or polyclonal antibodies, each of which have their own advantages and disadvantages. Monoclonal antibodies bear the obvious advantage of being a continuous, generally consistent, and potentially unlimited resource. The major disadvantage of a monoclonal antibody is its recognition of only one epitope, thus if the protein of interest can form a complex with other proteins (a highly probable scenario), it is possible that the epitope will be masked, decreasing the chances of pulling down all instances of the protein bound to DNA.

Polyclonal antibodies raised against the whole target protein get around this problem since the antibodies will recognize multiple epitopes, increasing the likelihood that there will be free epitopes, even in a protein complex, to interact with the antibody. However, polyclonal antibodies that are raised only to a subdomain or a peptide of the protein will recognize a reduced number of epitopes, bringing this type of antibody closer to the monoclonal condition. Another drawback of polyclonal antibodies is that only a limited quantity of serum, and therefore antibody, can be produced per batch (the amount is dependent on the species in which the antibody is raised). Since each new batch of serum may differ in its characteristics, each fresh batch of antibody must be tested as a new entity.

## Endogenous protein vs tagged protein

The majority of ChIP experiments currently rely on antibodies that recognize an endogenous protein expressed in cells of interest. However, a workable, validated antibody to a protein of interest cannot always be identified, despite extensive testing. To circumvent this problem, tagged versions of target proteins, expressed in relevant cells of interest, are sometimes used. This brings with it the advantage that well-characterized antibodies to epitope tags (e.g. V5, HA and His) are commercially available and therefore provide a consistent source of reagents.

Nonetheless we note that there are also disadvantages to this approach. One possible issue is that the tag may interfere with endogenous protein function, and potentially its interaction with DNA. It is hence advisable to compare the function of tagged and untagged proteins in suitable assays to confirm that they are functionally equivalent, before moving onto ChIP experiments. Additionally, in order to rule out non-specific binding of the anti-epitope tag antibody in the sample, a control ChIP reaction from identical cells, bar the expression of the tagged protein, should also be included (discussed in more detail below; ^24^).

In many situations, it is preferable to express the tagged protein at levels comparable to the endogenous protein, for instance by harnessing endogenous regulatory sequences to drive its expression (e.g. 28). This is because the overexpression of some, though not all, transcription factors may result in spurious binding ^29, 30^. Another, perhaps better, approach is to express the protein in cells mutant for the endogenous target, at levels that rescue the mutant phenotype (e.g. 31, 32). However, we note that in certain situations, over- or ectopic expression of DNA-binding proteins may produce the phenotype being studied, such as in lineage reprogramming or induced differentiation, in which case the non-endogenous binding of the factor gives useful information on the function and regulatory circuits controlled by that protein in such circumstances (e.g. 30, 33).

Recent technological advances in genome editing, such as TALEN and CRISPR/Cas9 technologies, now allow tags to be knocked into a specific locus to produce a fusion protein in many more different cell types and organisms than had previously been possible (e.g. 34, 35). Creating a tagged fusion protein obviates the issue of the protein being expressed exogenously at higher-than-endogenous levels, although not the disadvantage that the tag may interfere with protein function. Nevertheless, given that this approach potentially allows any protein to be tagged and ChIPed in any organism or cell type, its use seems set to increase massively in the future.

## Standard assays for ChIP antibody validation

In order for investigators to have confidence in an antibody’s specificity for the protein of interest, it is critical to have working standards and reporting guidelines ^23^, and this is as true for ChIP as for any antibody-based technique. It is prudent to take as much care as possible to ensure ChIP reagent fidelity in order to maximize the accuracy of research output. This has been particularly recognized by the ENCODE and modENCODE consortia, which have published guidelines for the standards required of ChIP-seq experiments for inclusion in their data pipeline ^24^. Other genome annotation consortia such as FAANG ( 36; http://www.faang.org/) and IHEC ( 37; http://ihec-epigenomes.net/) have also adopted such standards in order that data can be compatible and comparable, and we suggest that these validation assays are a useful toolkit for all researchers performing ChIP experiments. The guidelines typically suggest a two-step validation procedure: initially the antibody is tested in an immunoblot or immunofluorescence assay, followed by at least one secondary validation assay; these are described in brief below (see also Box 2). However, it should be noted that others have suggested these guidelines are not sufficiently stringent, and additional controls/validations may be required in certain experiments ^38^.

**Box 2. Suggested assays for ChIP antibody validation**

- Recombinant target proteins, or cell/nuclear lysates of relevant tissues may be immunoblotted with the antibody, and a strong immunoreactive band should be observed around the expected molecular weight of the target protein.

- A modification to the immunoblot assay is the ChIP-immunoblot. ChIP eluates containing antibody-target protein complexes are blotted and probed with antibody.

- The antibody may be tested in an immunofluorescent assay, where staining should be observed in the nuclei of target protein-expressing cells.

- Further immunoblot or immunofluorescence-based validation of antibody specificity may be carried out on cells/tissues in which the target protein has been knocked out or knocked down. The immunoreactive signal should be absent or greatly reduced.

- Proteins may also be translated *in vitro* or expressed in cells, and these samples may be tested in immunoblots or immunofluorescence, as an alternative method of evaluating reactivity.

- Immunoprecipitation with the antibody, followed by mass spectrometry-based sequencing, should identify a majority of the target protein in the pull down fraction.

- A second antibody to the target protein, or a tag-based pull down of tagged target proteins, may be used as an independent test. There should be good overlap of results between the different pull downs.

- A search for DNA motifs beneath ChIP peaks may be undertaken, and should enrich for the known binding consensus of the target protein.

- Peptide binding/competition assays may be performed to evaluate antibody specificity between the target epitope/protein and related proteins. ChIP-peptide methods may be used to quantitatively measure antibody specificity and affinity.

- Stable isotope labelling of amino acids in cell culture (SILAC) may also be used to quantitatively measure antibody specificity and affinity.

- To test for cross-reactivity against proteins related to the target, immunoblots or immunofluorescent experiments may be performed in cells/tissues in which the related protein(s) have been depleted. The antibody signal should not experience a reduction in this case.

- It may be cost-effective to first appraise antibodies in a medium/high-throughput pilot ChIP assay. If the antibody exhibits a reasonable ChIP signal, other validation steps may subsequently be undertaken.

## Primary validation

The primary validation most often employed is the immunoblot or western blot. This assay can be performed on cell or nuclear lysates, with the expectation that an immunoreactive band will be seen at the expected (or known) molecular weight for the protein of interest. In practice, it is likely the blot will reveal (many) other immunoreactive bands, which can suggest that the antibody recognizes other proteins in the sample. This may not be a problem if these other proteins are non-nuclear and hence not present in the chromatin sample being ChIPed; one way to test this is to perform the immunoblot with separate cytoplasmic and nuclear extracts. As a guideline, ENCODE accepts the immunoblot validation if the primary immunoreactive band makes up more than 50% of the blot signal. The immunoreactive band should be of the size expected for the protein, although if not, this will not necessarily rule out a specific signal since many factors, such as post-translation modifications, can affect the electrophoretic mobility of a protein. Especially in these situations, a secondary validation (see below) using cells with reduced or absent levels of the target protein will aid in determining whether the band represents the protein of interest.

Instead of a straight immunoblot assay, it can be advisable to perform a ChIP-immunoblot. In this version of the technique, the eluted protein-antibody complex from the ChIP is saved and run on a gel, then blotted and probed using the antibody against the protein of interest. This assay can be informative of whether the protein alone is pulled down in the ChIP reaction, or if other proteins are also in the eluate, suggesting cross-reactivity or non-specific binding. It can also be a useful guide as to whether the antibody will be successful in the full ChIP assay.

Immunoblots may however be challenging in some systems and with some antibodies, especially where transcription factors are be expressed at low levels. In this case, other methods may need to be considered to show that the antibody recognizes the protein of interest. For instance, the candidate protein can be overexpressed in the cells, or translated *in vitro*, and immunoblots performed on these protein samples ^39, 40^. Alternatively, an immunofluorescence assay may be used as a primary validation of the antibody ^24^, with the expectation that staining should be seen in the nuclei of cells in which the target is known to be expressed.

## Secondary validation assays

Given the caveats of the above primary assays, additional assays should be used to add support that an antibody is specific. These secondary assays address slightly different issues, and the more of these validation steps that can be taken, the better.

In order to further validate the specificity of the antibody immunoblots, immunofluorescence or ChIP assays can be carried out on samples from cells in which the target protein is knocked out or knocked down. In these experiments, the signal for the target protein should be absent or reduced in the mutant/knockdown cells compared to the control. As a guide, data is accepted into the ENCODE pipeline if the immunoreactive signal is reduced by at least 70% in immunoblot or immunofluorescence, or if the ChIP-seq (or ChIP-chip) signal is reduced by at least 50%, in the mutant or knocked down cells ^24^.

Immunoprecipitation followed by mass spectrometry-based sequencing can also be performed, with the expectation that the protein of interest will be identified in the sample ^24^. The presence of other proteins in the sample may not be problematic if these do not bind DNA. However, if other DNA-binding proteins are present these may represent non-specific binding of the antibody; conversely, they may merely represent other proteins that normally occur in a complex with the protein of interest on DNA ^41^. For the ENCODE project, the presence of other DNA-binding proteins was accepted, and samples entered the analysis pipeline, if the other DNA-binding proteins were present at a lower level than the target protein ^24^.

Another validation approach is to use multiple antibodies to the same protein of interest that target different parts of the protein (or protein complex) in a ChIP-seq assay. With this approach, a sizable overlap of protein-bound peaks in each ChIP should be seen; for instance, ENCODE has historically accepted an overlap of 75% of shared targets, although more recently another quality measure based on the irreproducible discovery rate has been employed ^24^. If other antibodies are not available then using an epitope-tagged version of the protein, and ChIPing with an anti-epitope tag antibody, is an alternative approach and should also give substantial overlap with the endogenous antibody (although caveats apply; see section on tagged proteins above). Indeed, even if an antibody has satisfied other secondary validations, it is still good practice to perform a ChIP with two different antibodies, when available.

Finally, a validation which applies specifically to ChIP-chip or ChIP-seq assays is motif enrichment. Since transcription factors recognize and bind specific DNA sequences, that sequence should be found under bound peaks by a motif search ^24^. ENCODE guidelines suggest searching for a known motif in a defined set of high-quality peaks, with data accepted if the motif is more than 4-fold enriched over all other accessible regions and is present in more than 10% of peaks ^24^. Alternatively if a *de novo* search for motifs reveals the known binding site, this can also corroborate that the antibody pulls down the protein of interest. However, it is worth noting that target proteins may not interact directly with DNA, in which case the lack of an enriched motif does not preclude the antibody being specific.

## Validation of histone modification antibodies

For anti-histone antibodies, additional validations to test the specificity and affinity of the antibodies are recommended. For instance in the ENCODE project, anti-histone antibodies were initially tested in immunoblots against a dilution series of whole-cell or nuclear extracts, and recombinant unmodified histones ^24, 26^. Although histones are highly conserved, antibody reactivity may vary between different species and so the antibodies were tested against lysates from each species used in the ChIP assays ^24^. In the case of histones, the guideline is that the specific histone band should make up at least 50% of the immunoblot signal and show at least 10-fold enrichment over any other individual band and the recombinant unmodified histone band ^24^.

Peptide binding or peptide competition assays, using histone tail peptides with particular modifications, are another class of methods to evaluate specificity of anti-histone antibodies ^24, 26^. In these tests, an enrichment in binding signal for the modification compared to other modifications should be seen; for ENCODE this enrichment was set at 10-fold ^24^. However, it should be noted that the lack of a signal does not rule the antibody out, since the antibody may not recognize a short peptide in an *in vitro* environment, but may still be able to bind to the modification in a ChIP assay. That being the case, an improvement to the assay may be the use of peptide ChIP, which allows a quantitative measure of specificity and affinity ^42^.

Other possible validations include mass spectrometry of immunoprecipitated samples as described above, with the target histone species accounting for at least 80% of the immunoprecipitated sample ^24^. An alternative, where resources allow, is mass spectrometry of immunoprecipitated samples after stable isotope labelling of amino acids in cell culture (SILAC). This method compares two samples that incorporate different isotopes of carbon or nitrogen (such as ^12^C, ^13^C, ^14^N or ^15^N), allowing the relative abundance of immunoprecipitated proteins to be determined in different samples, hence giving a quantitative measure of antibody specificity and affinity as described by Peach and colleagues ^43^. In addition, immunoprecipitation from cells depleted of or mutant for particular histone modifying enzymes, if available, may be used to validate that an antibody is specific to a particular histone modification ^24, 44^. Finally, once ChIP-seq data for the antibody is generated, binding profiles should be inspected for recognized patterns, such as for well-characterized modifications like H3K4me3 at transcription start sites; if binding is as previously established, this can also be taken as further corroboration that the antibody is specific and behaves as expected ^24^.

## Cross-reactivity with family members

Another issue to be aware of is potential cross-reactivity of the antibody with other proteins related to the protein of interest. For antibodies directed against members of a multi-gene family, it is best to use an antibody that recognizes regions unique to that particular family member of interest. Of course this is not always possible, either due to the nature of the protein, or the lack of suitable antibodies. Moreover, it may be that even if this precaution is taken, the antibody may still non-specifically bind to other family members of the target. Thus validation of the antibody should take related proteins into account.

Sequencing an immunoprecipitation reaction by mass spectrometry will give information on whether other family members are present in the sample (see secondary validation above). However, it may not always be possible to sequence the proteins by mass spectrometry, although it should be possible to take a candidate approach and test whether an antibody cross-reacts with related proteins that are expressed at the same time and in same place as the protein of interest. For instance, this can be tested in samples that are knocked down for the related family member, with the expectation that if the antibody spuriously recognizes this related protein, then the immunoreactive signal will be reduced or absent compared to the control. *In vitro* or *in vivo* translated proteins for related family members can also be used in immunoblots to ascertain whether the antibody cross-reacts with these related proteins (e.g. 39).

## Will your antibody work in a ChIP assay?

Unfortunately, even after all these assays have been performed for specificity, they are no guarantee that the antibody will have a high affinity for your protein of interest in a ChIP assay and give a good signal. For instance, Egelhofer and colleagues found that out of 80 anti-histone antibodies that had passed two validation assays (dot blot and immunoblot), 16 (20%) failed to produce a reliable ChIP-seq signal, despite 13 of those being advertised as ChIP-grade (see Supplementary Table 1 of 26). Similarly, Landt and colleagues reported that of 227 transcription factor antibodies that passed two ENCODE validation assays, only 44 (19%) also functioned in ChIP-seq assays ^24^.

As a considerable amount of effort can be expended in validating an antibody that subsequently fails to give a reliable signal in a ChIP assay, it may be best from a practical point of view to re-order the procedural sequence. For instance, it may be more efficient and cost-effective to initially test candidate antibodies in a medium throughput ChIP assay such as ChIP-string ^45^ or a pilot ChIP-seq assay ^46^. If a good ChIP signal is seen with a particular antibody (and especially if the known motif is identified underneath peaks of binding as a validation for specificity), then further validation steps, as suggested above, can be performed before continuing to use that antibody.

## Conclusions

ChIP is now a standard assay used to identify and study protein-DNA interactions, with its use greatly enhancing our understanding of how the genome is regulated in development and disease, and how it has evolved over time. However, its widespread use should not breed complacency in researchers, since the data generated can only be as good as the antibody used. We reiterate that it is critically important that ChIP antibodies be properly validated; tied to this, it is essential that the validations are properly reported on companies' antibody information sheets, and in research publications. We have highlighted a toolkit of possible measures that may be harnessed in validation studies, based on ENCODE guidelines, although we note that this list is not exhaustive and investigators should apply due consideration to the uniqueness of every experimental system and how validation may best be performed in each.
