# Supplementary material for: A ChIP on the shoulder? Chromatin immunoprecipitation and validation strategies for ChIP antibodies
Source: F1000Res. 2015 Jul 13;4:235. [Version 1] doi: 10.12688/f1000research.6719.1 (PMC4648227; doi:10.12688/f1000research.6719.1)
Supplement: Peer review history

# Peer review: Referee response for version 1

**DOI:** 10.5256/f1000research.7217.r10036

**Published:** 2015-10-12

**Authors:** Ana Pombo

**Affiliations:** 1 Epigenetic Regulation and Chromatin Architecture Group, Berlin Institute for Medical Systems Biology, Max Delbrück Center for Molecular Medicine, Berlin, Germany

The manuscript by Wardle and Tan provide an overview of technical concerns and strategies for quality control of antibody specificity in chromatin immunoprecipitation (ChIP) assays. The manuscript summarizes quality standards described previously by others, in particular those used in ENCODE and others.

I found the manuscript well written and very useful in general, although felt it could have considered more broadly the use of ChIP to map chromatin bound proteins or DNA modifications, beyond mapping transcription factors or histone modifications. ChIP is also used to map enzymatic activities that work on chromatin, such as DNA and RNA polymerases, chromatin remodelers and RNA processing machinery, DNA repair complexes, which can also be modified by post-translation modifications (PTMs). These other applications of ChIP may be beyond the intended focus of the manuscript, making the content feel incomplete on occasions. The authors may consider stating the focus of their review on ChIP for transcription factors and histone modifications, or alternatively expand the content to a broader coverage of the topic.

Major comments:

One main aspects that I felt could be improved is the fact that detection of post-translational modifications (PTMs) is not specific to histone modifications, and could work better in a separate section not associated with any kind of protein.

The second aspect was on specific remarks about whether ChIP is expected or not to only immunoprecipitate a single protein. 

Specific points:

a) "Monoclonal vs polyclonal"

It is argued that monoclonal antibodies have a drawback of only detecting one epitope. It could be pointed out that this can be an advantage in situations where researchers only wish to map a single epitope, as is the case for PTMs. In this case, a polyclonal seems to have only disadvantages, in particular the batch to batch unreliability.

b) "Primary validation" part 1

I was expecting to find the use of ELISA with specific peptides or recombinant proteins containing or not the epitope (e.g. peptides with/without PTM, or recombinant protein with/without modified or detected aminoacid(s)) discussed in this section, but it only appears later when discussing histones. 

c) "Primary validation" part 2

"This assay [western blot of ChIP chromatin] can be informative of whether the protein alone is pulled down in the ChIP reaction, or if other proteins are also in the eluate, suggesting crossreactivity or non-specific binding." 

Relatively few proteins will bind to chromatin alone, as the author acknowledges later in the manuscript, namely the subunit of a complex will immunoprecipitate with the other proteins of the same complex. I would not recommend a western blot of ChIPed chromatin as an assay of choice to judge the specificity of an antibody in ChIP. Alternative simpler methods can help judge the enrichment of chromatin bound by a given protein.

Subsequent section "The presence of other proteins in the sample may not be problematic if these do not bind to DNA." In line with the above comment, I would suggest either removing the comments that argue that only one protein can be immunoprecipitated for the ChIP to be specific, as this is in most cases an incorrect assumption.

d) "Validation of histone modification antibodies"

Several aspects of this section apply not only to histone modification antibodies, but to any PTMs to chromatin binding factors and enzymatic activities. The arguments for peptide binding etc should be put forward in a broader sense, not only for PTMs, but also to prove specificity of an antibody (mono- or polyclonal) to a given peptide.

Edits:

- Page 3. Use of word "ChIPed".

- Page 4. "immunofluorescent assay", I would suggest "immunofluorescence assay"

I have read this submission. I believe that I have an appropriate level of expertise to confirm that it is of an acceptable scientific standard.

# Peer review: Referee response for version 1

**DOI:** 10.5256/f1000research.7217.r10037

**Published:** 2015-09-15

**Authors:** Niall Dillon

**Affiliations:** 1 Gene Regulation and Chromatin Group, MRC Clinical Sciences Centre, Imperial College London, London, UK

The review by Wardle and Tan provides a clear and accessible treatment of the issue of antibody validation for chromatin immunoprecipitation (ChIP). The technical aspects of validating antibodes are covered effectively in the review. The authors also discuss the problems that can arise even with supposedly validated commercial antibodies – for example the fact that companies often change batches of polyclonal antibodies without revalidating them. This is an important problem affecting molecular biology and epigenetic studies and the review is a timely contribution to the discussion about how to address it.

I have read this submission. I believe that I have an appropriate level of expertise to confirm that it is of an acceptable scientific standard.
